# Integration, challenges, and future of artificial intelligence in critical care medicine: comprehensive applications from predictive models to clinical integration

**DOI:** 10.3389/fmed.2026.1874474

**Published:** 2026-07-22

**Authors:** Qijian Ji, Yingwei Wu, Mingkun Yang, Jitao Liu, Xiaofeng Zhang, Yijia Lin, Weihang Hu

**Affiliations:** 1 Department of Critical Care Medicine, Xuyi Clinical College, Yangzhou University, Huai’an, Jiangsu, China; 2 Department of Second Clinical Medical College, Zhejiang Chinese Medical University, Hangzhou, Zhejiang, China; 3 Department of Critical Care Medicine, The First People's Hospital of Aksu Region, Aksu, Xinjiang, China; 4 Affiliated Zhejiang Hospital, Zhejiang University School of Medicine, Hangzhou, Zhejiang, China; 5 Department of Critical Care Medicine, Zhejiang Hospital, Hangzhou, Zhejiang, China

**Keywords:** artificial intelligence, clinical integration, ethical challenges, intensive care medicine, multimodal data, predictive models

## Abstract

Artificial intelligence (AI) in intensive care units (ICUs) has advanced rapidly since 2018, with core applications in sepsis prediction, mechanical ventilation management, and acute kidney injury (AKI) early warning, utilizing machine learning and deep learning models on multimodal data such as vital signs and electronic health records to achieve high predictive accuracy, including AUROC values up to 0.96 for sepsis. Despite these developments, widespread clinical adoption faces significant challenges, including limited prospective multicenter validation, the “black-box” nature of algorithms, integration into clinical workflows, and ethical concerns regarding fairness and transparency, necessitating rigorous evaluation and multidisciplinary collaboration to translate AI into routine critical care practice.

## Graphical abstract

**Figure:**
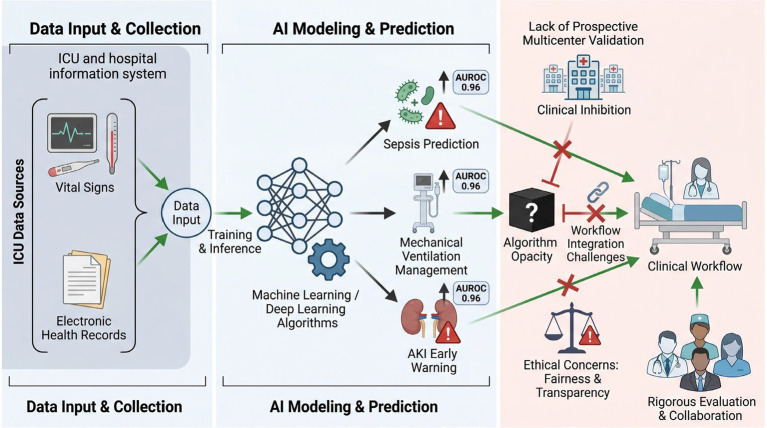

## 1 Introduction

The intensive care unit (ICU) represents one of the most data-intensive environments within healthcare systems, characterized by continuous monitoring and the generation of vast volumes of heterogeneous clinical data. This unique setting offers unparalleled opportunities for the application of artificial intelligence (AI) technologies aimed at enhancing patient care. Recent bibliometric analyses have documented a steep increase in AI-related research in intensive care medicine, particularly since 2018, with the United States and China leading contributions and a focus on neural networks, decision support systems, machine learning, and deep learning techniques (1). The ICU’s complex and dynamic clinical scenarios, involving rapid changes in patient status and multifaceted interventions, create a fertile ground for AI to support diagnosis, monitoring, prognostication, and workflow optimization. However, despite the proliferation of AI research, the translation of these advances into routine clinical practice remains limited, underscoring the need for comprehensive evaluations of AI integration in critical care (2).

AI applications in the ICU have evolved beyond early warning systems and sepsis prediction to encompass a broad spectrum of clinical domains. These include prediction models for mechanical ventilation weaning, acute kidney injury alerts, and personalized treatment recommendations tailored to individual patient physiology and disease trajectories. For instance, machine learning models such as the Hemodynamic Stability Index (HSI) have demonstrated superior predictive performance for hemodynamic instability compared to traditional single-parameter indicators, enabling earlier identification of patients at risk and potentially guiding timely interventions. Similarly, AI-driven models have been developed for real-time acuity assessment and prediction of life-sustaining therapy requirements, integrating diverse data streams including vital signs, laboratory results, and medication profiles, thereby enhancing clinical decision-making in rapidly evolving ICU contexts (3). Moreover, AI has shown promise in specialized ICU subfields such as cardiac critical care, where its capacity to analyze large datasets in real-time can assist in managing complex cardiovascular conditions (4). These advancements illustrate the expanding scope and sophistication of AI tools in critical care, moving toward comprehensive, multimodal data integration and personalized medicine.

Despite encouraging retrospective validation results, the widespread clinical adoption of AI in the ICU faces significant challenges. Many AI models remain at the experimental stage, with limited prospective multicenter validation and heterogeneous performance reporting. Issues such as data quality, interoperability, and the inherent “black-box” nature of many AI algorithms hinder clinician trust and acceptance. Furthermore, the ICU environment poses unique obstacles, including the need for real-time data access, integration into clinical workflows, and adaptation to individual patient responses. Ethical and legal considerations, such as fairness, transparency, and accountability, are paramount given the high-stakes nature of critical care decisions (5). Additionally, the lack of standardized endpoints and calibration metrics complicates the assessment and comparison of AI tools, while logistical barriers related to data sharing and governance limit the generalizability and robustness of predictive models. These multifaceted challenges necessitate a systematic and multidisciplinary approach to AI development, validation, and implementation in the ICU.

To bridge the gap between AI research and clinical practice, there is an urgent need for a comprehensive synthesis of the current state of AI applications in intensive care medicine, alongside a critical appraisal of the challenges and future directions. This includes a detailed examination of core AI applications such as predictive modeling, Clinical Decision Support Systems (CDSS), and multimodal data integration technologies, highlighting their respective strengths and limitations. Furthermore, addressing the ethical and legal frameworks governing AI use in critical care is essential to ensure equitable, transparent, and patient-centered deployment. Collaborative efforts involving clinicians, data scientists, regulatory bodies, and industry stakeholders are crucial to foster data sharing, harmonization, and the development of explainable AI models that can be seamlessly integrated into ICU workflows. Ultimately, advancing AI in critical care demands rigorous scientific evaluation balanced with pragmatic implementation strategies to realize its full potential in improving patient outcomes and healthcare efficiency.

## 2 Methods

To ensure methodological transparency and reproducibility in line with the SANRA (Scale for the Assessment of Narrative Review Articles) guidelines, we conducted a comprehensive literature search across three major databases: PubMed, Web of Science, and IEEE Xplore. The search was restricted to articles published between January 2015 and January 2026. Our search strategy utilized a combination of Medical Subject Headings (MeSH) and free-text keywords covering artificial intelligence (e.g., “artificial intelligence,” “machine learning,” “deep learning,” “generative AI,” “large language models,” “digital twins”) and critical care medicine (e.g., “intensive care,” “critical care,” “ICU,” “sepsis,” “mechanical ventilation,” “acute kidney injury”).

Given the narrative and scoping orientation of this review, our inclusion criteria focused on: (1) peer-reviewed original research articles; (2) systematic reviews and meta-analyses; and (3) established clinical consensus statements or guidelines. We explicitly excluded: (1) conference abstracts or proceedings lacking full empirical data; (2) non-English language articles; and (3) editorials or opinion pieces without original empirical data.

In addition to the primary database queries, we employed a manual reference-screening approach (snowballing) of the key retrieved articles to capture any highly relevant studies missed by the initial search algorithms. Consistent with the methodology of a narrative review, we did not conduct a formal quantitative risk-of-bias assessment. Instead, we performed a qualitative synthesis of the literature to identify broad thematic trends, evaluate current clinical applications, analyze translational barriers, and outline future directions for AI in critical care.

## 3 Results

### 3.1 Core application areas of artificial intelligence in intensive care

#### 3.1.1 Early prediction and management of sepsis

Sepsis remains one of the most lethal complications encountered in intensive care units (ICUs), characterized by its profound clinical heterogeneity, which complicates early detection and risk stratification. The complexity of sepsis arises from its multifactorial pathophysiology and variable patient responses, making timely diagnosis and intervention a critical yet challenging task. Artificial intelligence (AI) models have demonstrated significant promise in addressing these challenges by integrating diverse data streams such as vital signs, laboratory results, and electronic health records (EHRs) to facilitate earlier and more accurate sepsis detection. For instance, advanced AI frameworks employing gradient boosting machines and deep learning architectures have been developed and validated on large-scale datasets like MIMIC-IV, achieving outstanding predictive performance with area under the receiver operating characteristic curve (AUROC) values reaching 0.96. These models leverage temporal and multimodal data to identify subtle physiological changes preceding clinical deterioration, thereby enabling clinicians to initiate early interventions that can mitigate progression and improve outcomes. Moreover, AI-driven approaches allow for dynamic risk stratification, adapting to evolving patient conditions and heterogeneity inherent in sepsis presentations. Despite these advances, challenges remain in translating AI models into routine clinical practice, including the need for external validation across diverse populations, interpretability of model outputs to foster clinician trust, and integration within existing clinical workflows. Nevertheless, the growing body of research underscores AI’s transformative potential in enhancing sepsis management by shortening detection times and supporting personalized therapeutic strategies, ultimately aiming to reduce sepsis-related mortality in critical care settings (1, 6).

#### 3.1.2 Mechanical ventilation management and prediction of acute respiratory failure

The management of mechanical ventilation and the prediction of acute respiratory failure (ARF) are pivotal in optimizing treatment strategies within the ICU. Accurate forecasting of ARF progression and timely decisions regarding initiation and weaning from invasive mechanical ventilation are essential to minimize ventilator-associated complications and improve patient outcomes. Traditional scoring systems and biomarkers have shown limited predictive accuracy and often fail to capture the dynamic and multifactorial nature of respiratory failure. In contrast, machine learning (ML) models have emerged as powerful tools capable of integrating complex, longitudinal time-series data from physiological monitors, laboratory tests, and clinical notes to predict the need for invasive ventilation and optimal timing for extubation. These models utilize algorithms such as recurrent neural networks and ensemble methods to analyze temporal patterns and interactions among variables, providing real-time risk assessments that can guide respiratory support de-escalation. The success of these AI-driven models hinges on rigorous external validation, multidisciplinary collaboration with clinicians to ensure clinical relevance, and the development of implementation science frameworks that facilitate seamless integration into ICU workflows. Future advancements will likely focus on enhancing model interpretability and adaptability to diverse patient populations, thereby supporting personalized ventilation strategies that reduce ICU length of stay and ventilator-associated morbidity (7, 8).

#### 3.1.3 Prediction and early warning of acute kidney injury

Acute kidney injury (AKI) is a frequent and serious complication in critically ill patients, associated with increased morbidity, mortality, and prolonged ICU stays. Early identification of patients at risk for AKI is crucial to enable preventive measures that can avert irreversible renal damage. AI-based predictive models have been developed to leverage continuously updated patient data, including hemodynamic parameters, laboratory values, and medication profiles, to provide early warnings several hours before clinical onset. These models employ sophisticated machine learning techniques such as gradient boosting and deep neural networks to capture nonlinear relationships and temporal trends indicative of impending renal dysfunction. Validation studies using real-world ICU datasets have demonstrated high predictive accuracy, supporting the clinical utility of these tools. However, challenges remain in reducing false positive rates to prevent alarm fatigue and in tailoring model outputs to fit within clinical workflows without disrupting care delivery. Addressing these issues requires ongoing refinement of algorithms, incorporation of clinician feedback, and development of user-friendly interfaces that facilitate timely and actionable alerts. Ultimately, AI-driven AKI prediction holds promise for enhancing renal protection strategies and improving patient outcomes in critical care (1, 9).

#### 3.1.4 Hemodynamic monitoring and shock management

Hemodynamic instability is a common and life-threatening challenge in the ICU, often manifesting as shock with resultant organ hypoperfusion. AI-powered monitoring systems have been developed to continuously analyze complex physiological parameters such as blood pressure variability, heart rate dynamics, and arterial elasticity, enabling early detection of hemodynamic derangements. These systems integrate multimodal data streams, including waveform analyses and laboratory results, to predict episodes of hypotension and circulatory failure before clinical signs become apparent.

Cardiogenic shock-specific AI tools further enable real-time mortality risk stratification for patients with severe ventricular dysfunction (10). For patients supported by pVADs with tMCS, multimodal AI can predict device-related circulatory complications in advance (11). Cardiac surgical cohorts after cardiotomy also benefit from surgery-tailored machine learning prognostic models to forecast adverse hemodynamic events (12).

By providing real-time risk assessments and alerts, AI models empower clinical teams to implement preventive interventions that reduce the duration and severity of organ hypoperfusion. The integration of AI in hemodynamic monitoring also facilitates personalized management by identifying patient-specific patterns and responses to therapy. Despite promising results, widespread clinical adoption requires overcoming challenges related to data heterogeneity, ensuring model robustness across different monitoring devices, and addressing ethical considerations regarding automated decision support. Continued research and collaboration between engineers and clinicians are essential to refine these AI tools and embed them effectively within ICU practice (1, 13).

#### 3.1.5 Prognostic prediction and mortality risk stratification

Accurate prognostic prediction and mortality risk stratification are fundamental to guiding clinical decision-making and resource allocation in the ICU. AI models have demonstrated superior performance compared to traditional scoring systems such as APACHE and SOFA by analyzing large volumes of clinical, laboratory, and physiological monitoring data collected early during ICU admission. These models utilize advanced machine learning and deep learning techniques to identify complex patterns and interactions predictive of patient outcomes. Validation across multiple publicly available ICU datasets has confirmed their enhanced predictive accuracy and robustness. The application of these AI tools facilitates early identification of high-risk patients, enabling targeted interventions and informed discussions with patients and families regarding prognosis. However, the clinical utility of these models depends on their interpretability and transparency to ensure clinician trust and ethical application. Efforts in explainable AI are critical to elucidate model reasoning and support actionable insights. Furthermore, integrating prognostic models into clinical workflows requires careful consideration of usability and impact on care processes to maximize benefits (1, 14).

#### 3.1.6 Optimization of medical resources and workflow management

Beyond direct patient care, AI plays an increasingly vital role in optimizing ICU operational efficiency through enhanced resource management and workflow optimization. AI-driven predictive analytics can forecast ICU length of stay, readmission risk, and resource demands, thereby informing bed allocation, staffing schedules, and supply chain logistics. Machine learning algorithms such as XGBoost and random forests have been successfully applied to predict surgical case durations, post-anesthesia care unit resource utilization, and potential cancelations, contributing to improved perioperative management. Data-driven quality assessment frameworks enable continuous monitoring of ICU performance, identification of bottlenecks, and proactive risk mitigation. These applications not only enhance cost-effectiveness but also improve patient outcomes by ensuring timely access to critical care resources. Challenges include ensuring data privacy, interoperability among disparate hospital systems, and fostering acceptance among healthcare administrators and clinicians. Continued innovation and multidisciplinary collaboration are essential to harness AI’s full potential in ICU workflow management (15, 16).

#### 3.1.7 Large language models and automation of clinical documentation

Large language models (LLMs) represent a transformative advancement in AI with significant implications for automating clinical documentation in emergency and ICU settings. These generative AI systems can process and synthesize unstructured clinical text, enabling automated generation of medical notes, discharge summaries, and diagnostic reports, thereby alleviating the documentation burden on healthcare providers. By extracting actionable insights from electronic health records and clinical narratives, LLMs support clinical decision-making and enhance communication among care teams. However, deployment of LLMs in clinical environments necessitates addressing critical challenges, including model hallucinations—where AI generates plausible but incorrect information—data privacy concerns, and ethical responsibilities to maintain patient safety and trust. Ensuring transparency, rigorous validation, and integration with human oversight are paramount to realizing the benefits of LLMs while safeguarding against potential risks. As these technologies evolve, they hold promise for improving efficiency, accuracy, and quality of care documentation in critical care medicine (17, 18).

### 3.2 Multimodal data integration and AI model design

#### 3.2.1 Sources and characteristics of multimodal data

In the intensive care unit (ICU) setting, a wide variety of data modalities are generated, each providing unique insights into patient status and disease progression. These modalities include structured data such as vital signs and laboratory test results, unstructured textual data comprising clinical notes and nursing records, time-series waveform data like electrocardiograms (ECG) and arterial pressure waveforms, and imaging data including chest X-rays and computed tomography (CT) scans. Structured data are typically numerical or categorical and readily amenable to traditional statistical and machine learning analyses. Unstructured text data, encompassing physician notes, nursing documentation, and discharge summaries, contain nuanced clinical information but require natural language processing (NLP) techniques to extract meaningful features. Time-series waveform data capture continuous physiological signals, offering high temporal resolution that can reveal subtle changes in patient condition. Imaging data provide spatial and morphological information critical for diagnosis and monitoring.

Relying on a single data modality often fails to capture the complex, multifactorial nature of critical illness. For example, vital signs alone may not reflect underlying pathophysiological changes detectable in imaging or laboratory trends. Therefore, integrating multimodal data is essential to construct a comprehensive representation of patient status, enabling more accurate and robust AI models. Current research predominantly utilizes structured data and clinical text due to their relative accessibility and the maturity of analytical methods. However, waveform and video data remain underexploited despite their potential to enhance predictive performance. The heterogeneity and high dimensionality of multimodal ICU data pose challenges for data harmonization, feature extraction, and model training, necessitating advanced AI architectures capable of handling diverse data types and temporal dynamics. Overall, the integration of multimodal data sources is a critical step toward developing AI models that can more fully capture the complexity of critical care patients and improve clinical decision-making (1, 16, 19, 20).

#### 3.2.2 Multimodal fusion strategies and technical architectures

Multimodal data fusion in ICU AI applications involves combining heterogeneous data types to leverage complementary information and improve model performance. Among fusion strategies, intermediate fusion has emerged as the dominant approach, accounting for approximately 54% of studies. This strategy entails extracting modality-specific features through dedicated subnetworks and subsequently integrating these features at an intermediate stage within the model architecture. Intermediate fusion balances the preservation of modality-specific information with the benefits of joint representation learning, often outperforming early fusion (raw data concatenation) and late fusion (decision-level integration).

Technical architectures employed for multimodal fusion commonly include neural networks, Transformers, and attention mechanisms. Neural networks, particularly deep learning models, are adept at learning hierarchical feature representations from complex data. Transformers, with their self-attention mechanisms, enable effective modeling of long-range dependencies and cross-modal interactions, facilitating the integration of temporal and spatial information across modalities. Attention mechanisms further enhance interpretability and performance by weighting the contribution of each modality or feature dynamically based on relevance to the predictive task.

Empirical evidence demonstrates that multimodal AI models outperform single-modality counterparts in diagnostic and prognostic tasks within critical care. Specifically, multimodal models achieve an average area under the receiver operating characteristic curve (AUC) improvement of approximately 4.4%, reflecting enhanced discrimination capability. This performance gain underscores the value of capturing diverse physiological, clinical, and imaging information synergistically. Nonetheless, challenges remain in optimizing fusion architectures, managing missing or asynchronous data, and ensuring computational efficiency for real-time clinical deployment. Continued innovation in model design, including the incorporation of graph neural networks and multimodal Transformers, holds promise for advancing multimodal AI applications in the ICU (1, 6, 21).

#### 3.2.3 Explainability techniques and transparency requirements

The adoption of multimodal AI models in critical care hinges not only on predictive accuracy but also on model transparency and interpretability to foster clinical trust and facilitate informed decision-making. Approximately 14 distinct explainability techniques have been applied to multimodal AI models, aiming to elucidate the rationale behind model predictions. These techniques include feature attribution methods such as Shapley Additive exPlanations (SHAP), Local Interpretable Model-agnostic Explanations (LIME), attention visualization, and saliency mapping, which highlight influential features or data segments contributing to model outputs.

Explainable artificial intelligence (XAI) research in emergency and critical care medicine has grown rapidly, focusing on enhancing clinicians’ understanding of complex model decision processes. For instance, attention-based models provide insights into modality-specific contributions, while counterfactual explanations help identify minimal changes needed to alter predictions. Such transparency is crucial for identifying potential biases, validating model behavior, and ensuring alignment with clinical knowledge.

Despite these advances, significant limitations persist. There is a lack of standardized metrics and frameworks for evaluating explainability, complicating the comparison and validation of XAI methods. Moreover, prospective clinical validation of explainability tools remains scarce, limiting evidence of their utility in real-world settings. The absence of consensus on best practices for integrating explainability into clinical workflows further impedes widespread adoption. Addressing these gaps requires the development of unified evaluation standards, the incorporation of user-centered design principles, and rigorous prospective studies to assess the impact of explainability on clinical outcomes. Ultimately, enhancing transparency and interpretability is imperative to realize the full potential of multimodal AI in critical care and to ensure ethical, safe, and effective clinical integration (19, 22, 23).

### 3.3 AI in Clinical Decision Support Systems in intensive care

#### 3.3.1 Types and scope of Clinical Decision Support Systems (CDSS)

Clinical Decision Support Systems (CDSS) represent a pivotal interface for integrating artificial intelligence (AI) into intensive care units (ICUs), serving as the core platform through which AI-driven insights translate into actionable clinical guidance. These systems encompass a broad spectrum of functionalities, ranging from early warning alerts to sophisticated treatment recommendations, thereby addressing the multifaceted needs of critical care management. The diversity of CDSS types reflects the complexity of ICU environments: some systems focus on real-time monitoring and alerting for physiological deterioration, such as predicting the need for mechanical ventilation or vasopressor support, while others provide diagnostic assistance by interpreting imaging or laboratory data. For instance, AI models employing multivariate time series graph convolutional neural networks have demonstrated enhanced accuracy and interpretability in predicting ICU interventions, enabling clinicians to anticipate and prepare for critical therapeutic actions with improved confidence (24). Similarly, neural network-based CDSS trained on structured radiologic reports have augmented nonradiologist physicians’ ability to interpret bedside chest radiographs, thereby expanding diagnostic capabilities beyond traditional specialist domains (25). The scope of CDSS also extends to integrating multimodal data sources, such as combining imaging and clinical parameters through transformer-based architectures, which has been shown to improve diagnostic performance across multiple pathologies in ICU patients (26). Research hotspots in CDSS development emphasize seamless integration with clinical workflows, intelligent decision-making that supports precision ICU care, and validation of system effectiveness in managing complex patient populations. These systems are increasingly designed to not only predict adverse events but also to provide interpretable explanations of their recommendations, fostering clinician trust and facilitating informed decision-making. The evolution of CDSS in ICUs thus embodies a transition from isolated predictive models to comprehensive, context-aware platforms that support dynamic, patient-specific clinical decisions, ultimately aiming to enhance patient safety and outcomes.

#### 3.3.2 Clinical validation and effectiveness evaluation of CDSS

The clinical validation and effectiveness assessment of AI-based Clinical Decision Support Systems (CDSS) in intensive care settings remain critical yet challenging endeavors. Rigorous evaluation typically necessitates prospective, randomized controlled trials to establish causality and generalizability; however, the majority of current studies rely on retrospective analyses, limiting the strength of evidence for clinical adoption. Despite these constraints, several AI models integrated into hospital information systems have demonstrated promising capabilities in predicting sepsis severity, stratifying mortality risk, and providing early warnings for complications. For example, machine learning algorithms such as Light Gradient Boosting Machine (LightGBM) and neural networks have achieved high area under the curve (AUC) values in sepsis risk prediction among trauma patients, with some models deployed as online applications to facilitate real-time clinical use (27). Similarly, AI-driven prognostic models incorporating heart rate variability features have outperformed traditional scoring systems in mortality prediction, underscoring the potential of integrating physiological signals into CDSS (28). Nonetheless, key obstacles impede broader clinical validation and implementation. Data heterogeneity and lack of standardized formats across institutions hinder model generalizability, while overfitting and bias in AI algorithms can compromise reliability and fairness. Moreover, the absence of robust frameworks for human-machine collaboration limits the effective integration of AI recommendations into clinician workflows. Interpretability remains a pivotal concern; models that provide transparent, clinically meaningful explanations—such as those employing attention mechanisms or Shapley Additive exPlanations (SHAP)—are more likely to gain clinician acceptance (29). Additionally, challenges in artifact detection and data quality necessitate advanced preprocessing techniques, including neural network-based artifact recognition, to ensure input data integrity (30). Transfer learning approaches have shown promise in addressing data scarcity by leveraging pretrained models to improve outcome predictions in diverse ICU cohorts (31). Overall, while AI-enabled CDSS exhibit significant potential to enhance critical care decision-making, their clinical validation demands comprehensive, prospective studies, standardized data practices, and the development of integrative frameworks that foster synergistic human-AI collaboration to realize their full impact in ICU patient management.

### 3.4 Current AI applications in intensive care: comparison of data inputs, model types, validation status, and clinical implementation levels

#### 3.4.1 Comparison of data input sources and features

Artificial intelligence (AI) models applied in intensive care units (ICUs) rely on diverse data inputs that significantly influence their design, applicability, and generalizability. Different AI models utilize distinct types of data tailored to their clinical objectives. For instance, sepsis prediction models predominantly depend on time-series data of vital signs and laboratory results, capturing dynamic physiological changes indicative of infection and systemic response. These models often incorporate biomarkers such as immune-related proteins, as demonstrated by neural network analyses that identified key biomarkers like Programmed Death Ligand-1 and Myeloperoxidase associated with sepsis severity (32). Conversely, AI models for mechanical ventilation management emphasize respiratory mechanics parameters and waveform data, reflecting the need to monitor ventilatory status and optimize support settings.

Imaging-based AI applications, such as those interpreting bedside chest radiographs, primarily process DICOM-format medical images. A neural network trained on structured semiquantitative radiologic reports of ICU chest radiographs achieved diagnostic performance comparable to expert radiologists, enhancing nonradiologist physicians’ interpretation accuracy (25). Prognostic stratification models integrate multidimensional structured data, including demographics, comorbidities, and medication records, to capture patient heterogeneity and risk profiles. For example, sepsis risk prediction models have incorporated demographic and clinical variables alongside physiological scores to improve predictive accuracy (27).

The heterogeneity of data inputs directly impacts model applicability and external validity. Models trained solely on single-center electronic health record (EHR) data often suffer performance degradation when applied across institutions due to differences in data collection protocols, patient populations, and clinical practices (31). This limitation underscores the importance of multimodal data integration and multicenter datasets to enhance model robustness. Multimodal deep learning approaches that combine imaging and nonimaging data have demonstrated superior diagnostic performance compared to unimodal models, reflecting the complementary nature of diverse data types in ICU settings (26). Furthermore, the inclusion of high-resolution physiological waveforms, laboratory values, and clinical notes enables comprehensive patient monitoring and outcome prediction (33).

In summary, the choice of data inputs for ICU AI models is dictated by the clinical task, with sepsis prediction favoring time-series vital signs and labs, ventilation management relying on respiratory waveforms, imaging AI focusing on radiographs, and prognostic models integrating structured clinical data. The heterogeneity of these inputs necessitates careful consideration of data quality, standardization, and cross-institutional validation to ensure model generalizability and clinical utility.

#### 3.4.2 Evolution of model types and technical architectures

The landscape of AI models in intensive care has evolved markedly, with deep learning and ensemble learning emerging as dominant methodologies tailored to the complex, high-dimensional nature of ICU data. Deep learning architectures, including Long Short-Term Memory (LSTM) networks and Transformer models, excel in processing sequential and high-dimensional data such as time-series vital signs and physiological waveforms. For example, recurrent neural networks have been effectively employed for survival analysis and real-time acuity prediction, capturing temporal dependencies in patient data. Transformer-based models have further advanced multivariate multi-horizon forecasting of vital signs, demonstrating superior performance in capturing long-sequence dependencies and temporal feature dynamics (34).

Ensemble learning methods, such as XGBoost and LightGBM, have shown robust performance on structured tabular data, often outperforming traditional statistical models in tasks like sepsis prediction and mortality forecasting. These gradient boosting frameworks efficiently handle heterogeneous clinical variables and missing data, providing interpretable feature importance metrics that aid clinical understanding. Notably, in sepsis prediction among trauma patients, LightGBM models achieved optimal predictive performance, balancing accuracy and generalizability (27).

Emerging AI paradigms, including generative AI and reinforcement learning, are increasingly explored in ICU contexts. Generative models, exemplified by GPT series, facilitate clinical documentation automation and knowledge augmentation, potentially reducing clinician workload and enhancing decision support (35). Reinforcement learning approaches have been applied to optimize individualized treatment strategies, such as fluid resuscitation and vasopressor titration, by learning dynamic policies from longitudinal patient data (36). These methods address the challenge of sequential decision-making under uncertainty inherent in critical care.

A critical consideration in model architecture selection is the trade-off between predictive performance and interpretability. Deep learning models, while achieving high accuracy, often function as “black boxes,” limiting clinician trust and adoption. To mitigate this, interpretable architectures incorporating attention mechanisms, such as the double self-attention model, have been developed to elucidate variable importance over time, enhancing transparency without compromising performance (29). Conversely, traditional models like logistic regression and decision trees offer greater interpretability but may inadequately capture complex nonlinear relationships in ICU data.

In conclusion, ICU AI modeling has transitioned toward sophisticated deep learning and ensemble methods capable of handling diverse data modalities and temporal dynamics. The integration of interpretability techniques and exploration of generative and reinforcement learning models represent promising directions to balance accuracy, transparency, and clinical applicability.

#### 3.4.3 Validation status and clinical translation maturity

Despite the proliferation of AI models developed for intensive care applications, the majority remain confined to early validation stages, with limited progression toward clinical translation. Most AI models undergo retrospective, single-center validation, which restricts their generalizability and hinders widespread adoption. For example, sepsis early warning systems have predominantly been validated retrospectively within single institutions, with only a minority subjected to prospective cohort validation or randomized controlled trials (37). This limited external validation contributes to a clinical translation rate below 10%, reflecting the gap between model development and real-world implementation.

Models that have achieved multicenter prospective validation, such as acute kidney injury (AKI) prediction systems, demonstrate higher clinical integration, with partial embedding into hospital information systems (27). However, even these models face challenges related to data heterogeneity, workflow integration, and clinician acceptance. The lack of standardized validation frameworks exacerbates these issues, as inconsistent performance metrics and evaluation protocols impede comparative assessments and regulatory approval.

A significant barrier to sustained clinical performance is the absence of continuous monitoring and recalibration mechanisms post-deployment. Concept drift and shifts in data distributions over time can degrade model accuracy, necessitating adaptive validation strategies (38). Few studies report on ongoing performance surveillance or model updating, limiting the reliability of AI tools in dynamic ICU environments.

Furthermore, the scarcity of prospective interventional studies evaluating AI’s impact on patient outcomes constrains evidence-based adoption. While some models demonstrate promising predictive capabilities, their effect on clinical decision-making, workflow efficiency, and patient safety remains underexplored. This translational gap underscores the need for rigorous prospective trials, standardized reporting, and collaborative efforts to bridge development and deployment.

In summary, the validation landscape of ICU AI models is characterized by the predominance of retrospective single-center studies, limited external validation, and scarce prospective clinical trials. Advancing clinical translation requires standardized validation protocols, multicenter prospective studies, and robust post-deployment monitoring to ensure sustained performance and safety.

#### 3.4.4 Current clinical implementation levels and barriers to deployment

The clinical implementation of AI in intensive care units can be stratified into three progressive levels: experimental deployment, partial integration, and widespread adoption. Currently, most AI applications reside at the experimental deployment stage, often limited to pilot studies within single departments or units. For example, many sepsis prediction models and ventilator weaning support tools have been trialed in isolated ICU settings without broader system integration. This stage primarily serves to assess feasibility and initial performance but lacks scalability.

Partial integration involves embedding AI outputs into electronic health record (EHR) systems or clinical decision support platforms within institutions. Some AKI early warning systems and mechanical ventilation intervention predictors have achieved this level, facilitating clinician access to AI-generated alerts during routine care. However, these implementations often face challenges related to workflow disruption, alert fatigue, and user interface design, limiting their effectiveness and clinician acceptance.

Widespread adoption, characterized by standardized, cross-institutional AI deployment with regulatory approval and demonstrated clinical benefit, remains rare. Barriers to achieving this include poor compatibility with existing clinical workflows, where additional steps or clicks increase clinician burden, and high false positive rates leading to alarm fatigue and desensitization to alerts (37). Moreover, the lack of clear human-AI collaboration protocols impedes trust and effective use, as clinicians may be uncertain about how to interpret or act upon AI recommendations.

Successful implementation cases highlight key facilitators: transforming AI outputs into intuitive, actionable clinical prompts and preserving clinician autonomy in final decision-making. For instance, certain sepsis prediction systems that provide clear risk stratification alongside recommended actions have improved adoption rates (25). Additionally, integrating AI tools with clinician education and feedback mechanisms fosters acceptance and continuous improvement.

In conclusion, AI clinical implementation in ICUs is predominantly at early experimental stages, with limited partial integration and minimal widespread adoption. Overcoming barriers requires designing AI systems that seamlessly fit clinical workflows, minimize false alarms, and support effective human-machine collaboration to enhance clinician trust and patient outcomes.

### 3.5 AI in intensive care: ethical, legal, and regulatory challenges

#### 3.5.1 Data privacy and algorithmic bias

Algorithmic bias in artificial intelligence (AI) systems within intensive care units (ICUs) frequently originates from imbalanced population distributions in training datasets. Such disparities can result in significantly reduced predictive accuracy for minority groups, elderly patients, or specific disease subpopulations, thereby exacerbating existing healthcare inequalities. For instance, models trained predominantly on data from certain demographic groups may underperform when applied to underrepresented populations, leading to suboptimal clinical decisions and potential harm. This issue is compounded by the complexity of ICU patient profiles, where heterogeneity in clinical presentations demands robust and equitable model generalization. Data privacy regulations, such as the Health Insurance Portability and Accountability Act (HIPAA) in the United States and the General Data Protection Regulation (GDPR) in the European Union, play a critical role in safeguarding patient information. However, these regulations also impose substantial barriers to cross-institutional and cross-regional data sharing, which is essential for assembling diverse and comprehensive datasets that enhance model generalizability. The constraints on data interoperability and sharing limit the ability to train AI models on heterogeneous populations, thereby perpetuating algorithmic bias and restricting the improvement of predictive performance across diverse patient cohorts. To address these challenges, the implementation of “human-in-the-loop” or “human-machine collaboration” frameworks has been proposed. Such frameworks integrate computational efficiency with clinical expertise by incorporating manual review and oversight at the algorithm output stage. This approach not only enhances the safety and fairness of AI systems but also improves clinical acceptability by allowing clinicians to validate and contextualize AI-generated recommendations. Moreover, human oversight can help identify and mitigate biases that automated systems might overlook, fostering trust and accountability in AI-assisted ICU care. Overall, tackling data privacy concerns and algorithmic bias requires a multifaceted strategy that balances regulatory compliance, data diversity, and clinician involvement to ensure equitable and effective AI deployment in critical care settings (14, 39, 40).

#### 3.5.2 Regulatory approval and liability attribution

Most AI models intended for clinical use in ICUs are classified as “software as a medical device” (SaMD) and must undergo rigorous regulatory scrutiny before deployment. Current approval processes, however, are often lengthy and may not keep pace with the rapid evolution and iterative nature of AI technologies. This temporal mismatch poses a significant challenge, as AI models require frequent updates and retraining to maintain accuracy and relevance in dynamic clinical environments. The protracted regulatory timelines can delay the translation of innovative AI solutions into clinical practice, potentially hindering patient care improvements. Furthermore, the attribution of liability in cases where AI-generated erroneous recommendations lead to adverse clinical outcomes remains legally and ethically ambiguous. Responsibility may be diffused among AI developers, healthcare institutions, and clinicians, complicating accountability frameworks. This ambiguity raises concerns about the willingness of stakeholders to adopt AI tools and the establishment of clear guidelines for error management and risk mitigation. To ensure safe, compliant, and trustworthy AI integration in ICUs, the development of transparent, auditable, and continuously updatable governance frameworks is imperative. Such frameworks should facilitate ongoing model validation, performance monitoring, and compliance with evolving regulatory standards. They also need to delineate clear roles and responsibilities among developers, providers, and users to address liability concerns effectively. Establishing these governance structures is crucial for fostering clinical trust, ensuring patient safety, and enabling the sustainable adoption of AI technologies in critical care (8, 39).

### 3.6 Translational barriers and implementation strategies from research to clinical practice

#### 3.6.1 Insufficient model validation and generalizability limitations

A critical barrier impeding the translation of artificial intelligence (AI) models from research settings into routine intensive care unit (ICU) clinical practice is the prevalent insufficiency in rigorous model validation and the limited generalizability of these models. The majority of AI predictive models in critical care remain confined to retrospective, single-center validation studies, lacking robust prospective, multicenter clinical trials that are essential to establish clinical utility and safety. This limitation fundamentally restricts the clinical adoption rate of AI tools despite their promising retrospective performance. The heterogeneity of ICU environments, including variations in patient demographics, clinical practices, and data acquisition protocols, leads to significant differences in data distributions across centers. Such inter-institutional variability, coupled with imbalanced sample classes and temporal concept drift—where the underlying data characteristics evolve over time—substantially degrade the robustness and transferability of AI models when deployed in real-world clinical settings. For instance, models trained on data from one ICU may perform poorly when applied to another due to these distributional shifts. To address these challenges, establishing continuous performance monitoring mechanisms post-deployment is imperative. This includes real-time tracking of model accuracy and calibration to detect performance degradation. Additionally, implementing systematic periodic model retraining and updating strategies, leveraging new incoming data, can maintain and enhance model efficacy over time. Such adaptive learning frameworks ensure that AI systems remain relevant and reliable amidst evolving clinical environments. Collaborative efforts to conduct large-scale, multicenter prospective validation studies are also crucial to demonstrate the generalizability and clinical impact of AI models, thereby fostering clinician trust and regulatory approval. Without these rigorous validation and maintenance protocols, AI models risk obsolescence and may inadvertently compromise patient safety, underscoring the necessity of addressing these translational barriers comprehensively (1, 6, 8, 19).

#### 3.6.2 Clinical workflow integration and human-machine collaboration

Successful clinical implementation of AI tools in intensive care medicine hinges on their seamless integration into existing clinical workflows and fostering effective human-machine collaboration. AI systems that impose additional operational steps or disrupt established care routines risk increasing cognitive load on healthcare providers, thereby impeding adoption. The complexity and opacity of most artificial intelligence models—commonly referred to as “black boxes”—erode clinicians’ trust and disincline them from acting on algorithmic outputs, as the underlying reasoning driving model predictions lacks intuitive interpretability. This skepticism is a significant human factor barrier to AI acceptance in critical care settings. To overcome these challenges, AI design must embrace a human-centered approach that prioritizes usability and interpretability. Predictive outputs should be translated into clear, actionable insights presented through intuitive interfaces that align with clinicians”decision-making processes. For example, AI-driven risk scores or alerts should be contextualized with relevant clinical parameters and accompanied by explanations that elucidate the underlying reasoning. Moreover, fostering collaborative workflows where AI augments rather than replaces clinical judgment can enhance trust and facilitate adoption. Training and education programs aimed at improving clinicians”understanding of AI capabilities and limitations are also essential. By embedding AI tools within the natural flow of clinical care and promoting transparent, interpretable interactions, the synergy between human expertise and machine intelligence can be optimized, ultimately improving patient outcomes (16, 41, 42).

#### 3.6.3 Economic costs and unequal resource allocation

The deployment of AI systems in intensive care units is often constrained by substantial economic costs and disparities in resource availability, which pose significant challenges to equitable implementation. High initial investments in specialized hardware, software licensing, and ongoing maintenance expenses limit the feasibility of AI adoption, particularly in resource-limited healthcare settings. This financial barrier is especially pronounced in low- and middle-income countries (LMICs), where infrastructural deficiencies and funding shortages exacerbate existing healthcare inequities. Consequently, the uneven distribution of AI technologies risks widening the gap in critical care quality between well-resourced and under-resourced institutions, potentially perpetuating health disparities. To mitigate these issues, developing cost-effectiveness evaluation models is vital to quantify the economic and clinical benefits of AI interventions relative to their costs. Such analyses can inform strategic allocation of limited resources and justify investments in AI technologies that demonstrate favorable value propositions. Furthermore, adopting tiered implementation strategies tailored to the economic context of healthcare facilities can facilitate scalable and sustainable AI integration. For instance, lightweight AI models optimized for lower computational requirements or cloud-based AI services can reduce infrastructure burdens. Policymakers and stakeholders should also prioritize funding mechanisms and partnerships that support AI accessibility in underserved regions. Addressing economic and resource allocation challenges is essential to ensure that AI-driven advances in critical care medicine benefit diverse patient populations globally, promoting health equity (8, 16).

#### 3.6.4 Challenges of implementation failure and workflow disruption

Real-world deployment of AI systems in intensive care units frequently encounters implementation failures manifesting as workflow disruptions, alarm fatigue, and progressive model performance deterioration, which collectively hinder routine bedside application. Integration of AI tools may inadvertently interrupt established clinical processes, leading to inefficiencies and clinician frustration. Excessive or poorly calibrated AI-generated alerts contribute to alarm fatigue, diminishing the responsiveness of healthcare providers to critical warnings and potentially compromising patient safety. Additionally, AI models may experience performance decay over time due to changes in clinical practice patterns, patient populations, or data acquisition methods, necessitating ongoing recalibration and validation. Beyond technical challenges, regulatory uncertainties surrounding AI approval, interoperability limitations among disparate health information systems, and ambiguous legal liability frameworks further complicate the transition from research prototypes to clinical deployment. These institutional and systemic barriers form complex obstacles that impede the seamless adoption of AI in critical care. To surmount these challenges, comprehensive implementation frameworks encompassing technical, organizational, and regulatory dimensions are required. Iterative optimization cycles involving multidisciplinary collaboration among clinicians, data scientists, engineers, and administrators can refine AI integration and minimize workflow disturbances. Establishing clear regulatory pathways and legal guidelines will provide necessary governance and accountability. Moreover, fostering interoperability standards and ensuring compatibility with existing electronic health record systems will facilitate smoother AI incorporation. Through such holistic and coordinated strategies, the core barriers to routine bedside AI application in intensive care can be progressively overcome, enabling the realization of AI”s full clinical potential (8, 42).

### 3.7 Generative artificial intelligence’s emerging role in intensive care

#### 3.7.1 Applications of large language models in clinical documentation and decision-making

Large language models (LLMs), exemplified by the GPT series, have demonstrated remarkable capabilities in processing and extracting critical information from unstructured clinical text, thereby offering transformative potential in intensive care unit (ICU) workflows. These models excel at synthesizing free-text data such as physician notes, nursing records, and diagnostic reports to generate coherent, accurate clinical documentation including discharge summaries and progress notes. This automation not only enhances clinical efficiency by reducing documentation burden but also improves the consistency and completeness of records, which are vital for continuity of care in the ICU setting (43, 44). In perioperative medicine, LLMs extend their utility beyond documentation to personalized risk stratification and real-time integration of diverse data streams, including vital signs, laboratory results, and imaging findings. By assimilating this multimodal information, LLMs can support dynamic clinical decision-making, enabling tailored interventions and facilitating clearer communication between clinicians and patients or their families (45, 46). However, despite these promising applications, overreliance on LLMs poses significant risks. The inherent limitations of LLMs—such as susceptibility to generating plausible but incorrect information (“hallucinations”), potential biases embedded in training data, and lack of transparency in reasoning—may compromise patient safety and undermine the humanistic aspects of care (47, 48). Therefore, it is imperative to establish rigorous governance frameworks that define appropriate use cases, incorporate human oversight, and ensure continuous validation of LLM outputs within clinical workflows. Such frameworks should emphasize ethical considerations, data privacy, and the preservation of patient-centered care to harness LLMs’ benefits while mitigating risks in critical care environments (49, 50).

#### 3.7.2 Multimodal generative models and the concept of “digital twins”

The concept of digital twins—dynamic, virtual representations of patients’physiological systems—has gained traction as a frontier in critical care, promising to revolutionize disease modeling and therapeutic optimization. By integrating real-time patient data streams, including hemodynamic parameters, laboratory values, and imaging, digital twins simulate the progression of critical illness and predict responses to interventions, thus enabling precision medicine in the ICU (51). Recent advances in generative artificial intelligence, particularly the fusion of large language models (LLMs) with vision-language models (VLMs), have expanded the capabilities of digital twins to process and interpret heterogeneous data modalities simultaneously. This multimodal integration facilitates comprehensive patient state representation, allowing for nuanced clinical forecasting and personalized treatment planning (52). Despite these advances, significant challenges remain before digital twin technology can be routinely implemented in critical care. Real-time data integration demands robust interoperability among disparate electronic health record systems and medical devices, while ensuring data quality and minimizing latency is critical for timely decision support (2). Moreover, the interpretability of complex multimodal models is essential to foster clinician trust and facilitate actionable insights, necessitating the development of explainable AI techniques tailored to critical care contexts (53). Finally, rigorous clinical validation through prospective, multicenter studies is required to establish safety, efficacy, and generalizability of digital twin applications. Addressing these challenges will be pivotal to realizing the full potential of multimodal generative AI and digital twins in enhancing precision and responsiveness of critical care interventions.

### 3.8 Future directions and roadmap

#### 3.8.1 From single-center to multi-center joint validation

The future of artificial intelligence (AI) in intensive care medicine critically hinges on transitioning from predominantly single-center, retrospective studies to robust multi-center, prospective clinical trials. Current AI models often suffer from limited generalizability due to the narrow scope of data sources, which restricts their applicability across diverse patient populations and healthcare settings. To overcome these limitations, future research must prioritize the establishment of standardized frameworks for model validation that encompass heterogeneous datasets from multiple institutions. Such frameworks would ensure that AI algorithms are rigorously tested for reproducibility, robustness, and clinical utility in real-world scenarios. The integration of federated learning techniques represents a promising avenue to facilitate collaborative model training across institutions while preserving patient privacy and data security. Federated learning enables decentralized learning by allowing models to be trained locally on institutional data and then aggregated centrally without sharing raw data, thus enhancing model generalization and robustness without compromising confidentiality. Moreover, adopting standardized interoperability data formats, such as Fast Healthcare Interoperability Resources (FHIR), will be instrumental in enabling seamless data exchange and model deployment across disparate electronic health record (EHR) systems. This standardization will not only facilitate the portability of AI models but also streamline their integration into clinical workflows, thereby accelerating clinical adoption. Collectively, these strategies will address the current challenges posed by single-center studies and pave the way for AI models that are validated across diverse clinical environments, ultimately enhancing their reliability and impact in critical care practice (1, 6).

#### 3.8.2 Interdisciplinary collaboration and AI literacy development

The successful integration of AI into critical care medicine necessitates a concerted interdisciplinary effort involving clinicians, data scientists, ethicists, and policy makers. Such collaboration fosters an ecosystem of co-innovation where clinical insights guide algorithm development, and technical expertise informs clinical applicability and ethical considerations. Embedding AI literacy into critical care training programs is paramount to empower clinicians with the skills to critically appraise AI model outputs, understand their limitations, and actively participate in the iterative process of algorithm refinement. This educational initiative should encompass foundational knowledge of machine learning principles, interpretability techniques, and the ethical implications of AI deployment. Furthermore, interdisciplinary teams are essential for establishing responsible AI governance frameworks that prioritize patient welfare, transparency, and fairness. These frameworks should address issues such as bias mitigation, accountability, and equitable access to AI technologies, ensuring that innovations serve clinical needs rather than purely technological advancement. By cultivating AI literacy and fostering collaborative governance, the critical care community can harness AI as a trustworthy adjunct to clinical decision-making, promoting patient-centered care and ethical stewardship of emerging technologies (39, 42, 54).

#### 3.8.3 Dynamic, adaptive, and continuous learning systems

Future AI systems in intensive care units (ICUs) must evolve beyond static models to dynamic, adaptive frameworks capable of continuous learning from real-time clinical data and feedback. Such systems should accommodate concept drift and shifts in data distributions inherent in evolving patient populations and clinical practices, thereby maintaining optimal predictive performance over time. Incorporating mechanisms for online learning and model updating will enable AI tools to adapt to local data characteristics and emerging clinical patterns, enhancing their relevance and accuracy. Real-time data stream processing and low-latency inference are critical technical requirements for bedside deployment, necessitating the development of efficient computational architectures and edge computing solutions that can operate within the constraints of ICU environments. Importantly, AI should be positioned as an augmentative tool that enhances clinical judgment rather than replacing it, preserving the humanistic and ethical dimensions of medical care. Maintaining clinician oversight ensures that AI recommendations are contextualized within the broader clinical picture, safeguarding against overreliance on algorithmic outputs. This human-in-the-loop paradigm supports ethical responsibility and patient-centeredness, ensuring that AI serves as a facilitator of informed clinical decisions rather than an autonomous decision-maker (19, 41, 55).

## 4 Conclusion

The integration of AI in critical care medicine has evolved from theoretical concepts to sophisticated, multimodal systems targeting high-mortality conditions such as sepsis, acute kidney injury, and respiratory failure. By synthesizing diverse clinical data streams, AI has demonstrated immense potential for early detection, dynamic risk stratification, and personalized decision support. However, the translation of these research prototypes into routine bedside practice remains severely bottlenecked. Current clinical implementation is impeded by a lack of prospective multicenter validation, the “black-box” nature of algorithms, workflow disruptions, and substantial ethical, legal, and economic barriers.

To bridge this translational gap, future efforts must shift from isolated, retrospective modeling toward rigorous, prospective clinical trials. XAI, implementing federated learning to ensure data privacy, and developing human-in-the-loop collaborative frameworks are crucial for fostering clinician trust. Furthermore, establishing standardized regulatory governance and addressing the economic constraints in resource-limited settings are essential for equitable deployment. Ultimately, realizing the transformative potential of AI in the ICU requires a concerted, interdisciplinary collaboration to ensure that technological innovations safely and effectively augment—rather than replace—clinical expertise at the bedside.
